# The Role of the Tumor Microenvironment in Neuropilin 1-Induced Radiation Resistance in Lung Cancer Cells

**DOI:** 10.7150/jca.28163

**Published:** 2019-07-08

**Authors:** Zhuo Dong, Haiyang Zhang, Xinkou Gong, Wei Wei, Yahui Lv, Zhiyuan Chen, Rui Wang, Junxuan Yi, Yannan Shen, Shunzi Jin

**Affiliations:** 1 NHC Key Laboratory of Radiobiology, School of Public Health, Jilin University, Changchun, 130021, China; 2 Department of Prosthodontics Dentistry, The Stomatology Hospital of Jilin University, Changchun, 130021, China; 3 Department of Radiology, The 2 nd Hospital of Jilin University, Changchun, 130021, China

**Keywords:** Tumor microenvironment, NRP1, radiation resistance, three-dimensional (3D) culture, epithelial-mesenchymal transition (EMT)

## Abstract

**Background**: Neuropilin 1 (NRP1) is a pleiotropic receptor which can interact with multiple ligands and their receptors. It plays an important role in the process of axonal growth, angiogenesis, tumor metastasis and radiation resistance in endothelial cells and some tumor cells. Interaction of stromal and tumor cells plays a dynamic role in initiating and enhancing carcinogenesis, and has received considerable attention in recent years.

**Material and Methods**: In this study, A549 lung cancer cell lines with different NRP1 expression levels were constructed *in vitro*, a two-dimensional (2D), three-dimensional (3D) co-culture system and tumor-bearing model was established in SCID mice. Western blot, qRT-PCR, immunofluorescence, cytometric bead array and flow cytometry were used to investigate the effect of the tumor microenvironment in NRP1-induced lung cancer cell radiation resistance.

**Results**: In 2D or 3D co-culture system, NRP1 could be regulated inflammatory factors such as TNF, IL-6 IL-8 and IL-17 and the related chemokines MCP-1, IP-10 and RANTES in the tumor microenvironment, which in turn induced radiation resistance in lung cancer cells. In addition, different expression levels of NRP1 in 2D, 3D culture systems and tumor-bearing models were able to significantly regulate cell phenotype, proliferative capacity, epithelial-mesenchymal transition (EMT) and the radiation resistance of A549 cells.

**Conclusion**: Our results verified that NRP1, inflammatory factors, chemokines and related signaling pathways, which affect the transformation of related cell components and thus lung cancer cell immune tolerance and migratory ability, all play an important role in radiation resistance.

## Background

Lung cancer is currently the most frequent malignant tumor in the world 1. The treatment of lung cancer is mainly through surgical treatment, radiotherapy and chemotherapy to cure or control the disease 2. As the main treatment method for cancer, radiotherapy has become an effective therapy 3; however, even after advanced radiotherapy, some patients still show radiation resistance and suffer subsequent cancer recurrence and metastasis 4, 5. Therefore, the radiation resistance of tumor cells remains a major obstacle in effective tumor radiotherapy.

Neuropilin 1 (NRP1) is extensively expressed in tumor vasculature, where its overexpression has been associated with tumor progression and poor clinical outcome 6. NRP1 is not only associated with tumor malignancy, but can also enhance the radiation resistance of tumors through the activity of vascular endothelial growth factor, semaphorin and other factors which influence radiation resistance of tumor cells 7, 8. With the development of cell biology technology has come the understanding that cellular processes such as cell proliferation, differentiation and apoptosis are all influenced by the extracellular microenvironment 9, 10. The tumor microenvironment is a complex system containing a variety of stromal cells which act through complex signaling pathways to secrete a variety of inflammatory cytokines, chemokines and angiogenic factors that accelerate tumor development 11-13. However, little is known about the mechanisms of NRP1-induced tumor cell radiation resistance in the microenvironment at different stages of the tumor.

Unfortunately, two-dimensional (2D) culture models cannot replicate the complexity of the tumor microenvironment. The development and application of advanced three-dimensional (3D) cell culture systems has overcome many of the limitations of traditional 2D monolayer cell culture systems by mimicking more closely the complex cellular heterogeneity and interactions that influence tumor microenvironmental conditions 14. Therefore, the remarkable plasticity of cancer cells under different experimental conditions can be easily reproduced by 3D cultures, which allow for re-establishment *in vitro* of crosstalk among neighboring cells and their surrounding stroma and enable a better understanding of the molecular and cellular mechanisms affecting tumors 15, 16. In this study, we first constructed an A549 cell line with differential expression of NRP1 *in vitro*, and established a 2D or 3D co-culture system to mimic the *in vivo* microenvironment, and then further validated the 3D tumor model by constructing a mouse tumor-bearing model *in vivo* to study the role of NRP1 in radiation-induced lung cancer cell radiation resistance in inflammatory and migratory microenvironments. The aim was to provide a new theoretical and experimental basis for radiotherapy in clinical lung cancer.

## Materials and Methods

### Cell lines and culture

The human lung adenocarcinoma A549 cell line, the Jurkat immortalized line of human T lymphocyte cells and human lung fibroblast cells HLF-1 were obtained from the Type Culture Collection of the Chinese Academy of Sciences (Shanghai, China,). Cell lines were cultured in RPMI-1640 medium (Gibco, Grand Island, USA) or DMEM (Gibco) supplemented with 10% (vol/vol) fetal bovine serum (HyClone, Waltham, USA) and 1% penicillin-streptomycin at 37℃ in a humidified atmosphere of 5% CO_2_.

For peripheral blood lymphocyte separation, lymphocyte separation medium (Organon Teknika, Durham, NC, USA) was aseptically transferred into a centrifuge tube. Human blood collected in anticoagulant and RPMI-1640 medium were mixed 1:1 and slowly added to the centrifuge tube, followed by centrifugation at 1500 g for 20 min at room temperature. The supernatant contained four layers; the lymphocyte layer and half of the LSM were withdrawn and washed twice with an equal volume of RPMI-1640 to obtain lymphocytes. Fresh human blood was obtained from volunteers at the First Affiliated Hospital of Jilin University (Changchun, China) and used within 8 h. The study was approved by the Medical Ethics Committee of the First Affiliated Hospital of Jilin University, and written informed consent was obtained from all volunteers.

The A549 cell model of radiation-resistance (A549RR) used cells in the logarithmic growth phase. A549 cells were digested with trypsin and counted, then inoculated at 2×10^4^ cells in cell culture flasks (75 cm^2^) and exposed to 6 Gy X-ray irradiation after cell adherence. Clones which formed 10-12 days later were digested and seeded at 2×10^4^ cells in new cell culture flasks. After adherence, the cells were again irradiated with 6 Gy X-rays, the entire process was repeated 5 times with a total radiation dose of 30 Gy. Clonal cells which formed after the last irradiation were considered radiation-resistant cells. To determine the success of the model, the cell proliferation rate and colony formation rate were determined after exposure to 10 Gy X-ray radiation. The A549 cell model of NRP1 interference (NRP1^Low^A549) was established and frozen in accordance with a previously described method from our group 7.

### 2D and 3D cell co-culture models

A549 cells in logarithmic growth phase were seeded at 3×10^5^ cells into the top chamber of each well in 24-well Transwell plates (Corning, Corning, NY, USA) and were allowed to adhere for 10 h. Extracted human peripheral blood lymphocytes or HLF-1 cells were then inoculated at 1.5×10^5^ cells into the bottom chamber of the wells to establish a 2D co-culture model. After 2D co-culture in a cell incubator for 48 h, the irradiation group was exposed to 10 Gy X-ray radiation and the cell supernatants from irradiated and control cells were collected 48 h later for subsequent experiments.

To prepare the 3D cell culture model, Matrigel stock solution at 10.6 mg/ml was allowed to dissolve overnight at 4°C. Cells in the exponential growth phase were digested in 0.25% trypsin and diluted with serum-free medium to a density of 1×10^6^ cells/ml, then added to an equal volume of Matrigel in an ice bath and quickly inoculated in 24-well plates at 200 μl per well. The cells were then incubated for 30 min at 37°C, followed by the addition of 1 ml complete medium and incubation at 37℃ at 5% CO_2_ for use in the next experiment.

The cell 3D co-culture model was established as described earlier. The cell lines A549, A549RR or NRP1^Low^A549 (2×10^5^ cells per well) in Matrigel were inoculated into the top chamber of 24-well Transwell plates and Jurkat or HLF-1 cells were inoculated into the bottom chamber at 1×10^5^ cells per well to establish co-culture 3D models of A549, A549RR and NRP1^Low^A549 cells with Jurkat or HLF-1 cells. After 3D co-culture in a cell incubator for 48 h, the irradiation group was exposed to 10 Gy X-ray radiation. After incubation for 3 days, the 3D co-cultured medium was collected and centrifuged to remove cellular debris, and the supernatants were frozen at -80°C. Co-cultured HLF-1 cells were collected for immunofluorescence or qRT-PCR analysis of α-SMA, TGF-β and Smad7.

### Animals and Mouse tumor-bearing model

SCID mice were purchased from Beijing Huafukang Biotechnology Co., Ltd. (quality Certificate No. 11401300031253), 35-42 days old, weighing 18±2 g, all male, aseptically raised in the isolator (PR model, Suzhou Suhang Technology Equipment Co., Ltd., China). A total of 36 mice were randomly assigned to 6 different groups: A549 group, A549-IR group, NRP1^Low^A549 group, NRP1^Low^A549-IR group, A549RR group and A549RR-IR group.

A549, NRP1^Low^A549 or A549RR cells were suspended to a concentration of 1×10^7^ cells/mL, and 1 mL syringe was used to slowly inoculate 0.1 mL of the suspended cells into the hind leg flank subcutaneous of each nude mouse. At 5 days after inoculation, the inoculation sites were observed, and the tumor mass sizes were initially recorded. After about two weeks, when the tumor reached an apparent mass of 50 g (i.e., 50 mm^3^ ), the mice of irradiated group were then exposed to X-rays, and received 20 Gy of irradiation, and then the weight of the mice, long diameter and the short diameter of the subcutaneously implanted tumor were recorded every other day. 7 days after radiation the nude mice had been sacrificed, tumor volumes were calculated as V = length×width^2^/2, and the tumor masses were excised and weighed. The NRP1 protein expression in tumours was then detected by immunofluorescence analysis.

### Irradiation protocol

Cells were sham-irradiated or exposed to ionizing radiation (IR) at 10 Gy which was delivered at the dose rate of 0.341 Gy/min and a source skin distance of 60 cm by an X-ray generator (Model X-RAD320iX; Precision X-Ray, Inc., North Branford, CT, USA).

Mice were sham-irradiated or partial exposed to IR at 20 Gy which was delivered at the dose rate of 1.0 Gy/min and a source skin distance of 70 cm by an X-ray generator.

### H&E staining analysis

Tumors tissues were removed and fixed with 4% paraformaldehyde for > 24 h. After fixation, the tissues were trimmed, placed in dehydration boxes, and dehydrated with different concentrations of alcohols and xylene; after which, the tissues were embedded in an embedding machine. After trimming the excess paraffin, the tissues were cut into 4 μm thick sections that were de-paraffinized to water. The cell nuclei were stained with Harris hematoxylin for 4-8 min, differentiated with 1% hydrochloric acid/alcohol solution, turned blue with 0.6% ammonia/water solution, and then washed with water. Next, the sections were stained with eosin for 2-3 min; after which, they were dehydrated with alcohol and sealed with neutral gum. The stained sections were observed under a microscope and photographed for analysis. After staining, the nucleus developed a blue color and the cytoplasm appeared red.

### Quantitative real-time polymerase chain reaction (qRT-PCR)

Total RNA from cells was isolated with TRIzol (Invitrogen, Carlsbad, CA, USA) according to the manufacturer's protocol and reverse transcribed to generate cDNA (PrimeScript RT-PCR kit; TaKaRa, Dalian, China). qRT-PCR was performed using the SYBR Green assay (TaKaRa) with *GAPDH* as an internal control. cDNA levels were quantified by real-time PCR with the 7300 Real-Time PCR System (Applied Biosystems, Foster City, CA, USA). The sequences of the primers were presented in Supplementary Table 1. All qRT-PCR assays were performed in duplicate. Relative quantification of gene expression was calculated using the 2^-ΔΔCT^ method 17.

### Cytometric bead array (CBA)

Cell supernatants were collected after 5 min centrifugation of cells at 1500 x g. The BD^™^ CBA Human Inflammatory Cytokines Kit (BD Biosciences, San Jose, CA, USA) was used to quantitatively measure interleukin (IL)-8, IL-1, IL-6, IL-10, tumor necrosis factor (TNF) and IL-12p70 protein levels in a single sample. The BD™ CBA Human Chemokine Kit was used to quantitatively measure CXCL8/IL-8, CCL5/RANTES, monokine induced by interferon-γ (CXCL9/MIG), monocyte chemoattractant protein-1 (CCL2/MCP-1) and interferon-γ-induced protein-10 (CXCL10/IP-10) levels in a single sample, following the manufacturer's protocol with minor modifications. The concentration of serum cytokines was quantified using Cell Quest Pro and CBA software (BD Biosciences) on a FACS Calibur flow cytometer (BD Biosciences).

### Protein extraction and western blot analysis

Cells were harvested by lysis in radio immunoprecipitation assay buffer (RIPA, Beyotime, Shanghai, China) for 30 min. The protein concentration was determined with BCA assay (Beyotime biotechnology, China). Protein lysates were then separated with sodium dodecyl sulfate-polyacrylamide gel electrophoresis and transferred onto nitrocellulose membranes (Millipore, Billerica, MA, USA). For western blotting, the membranes were blocked in 5% fat-free dry milk solution in phosphate buffered saline (PBS) and then incubated with primary antibodies anti-NRP1 (1:1000; Abcam, Cambridge, MA, USA), anti-α-SMA (1:1000; Cell Signaling Technology, Danvers, MA, USA), anti-TGF-β (1:1000; Cell Signaling Technology), anti-Smad7 (1:500; Santa Cruz, CA, USA), anti-Smad2 (1:1000; Cell Signaling Technology), anti-Smad3 (1:1000; Cell Signaling Technology), and anti-GAPDH (1:5000; Proteintech Group Inc., Chicago, IL, USA), followed by subsequent incubation with secondary antibody from the Super Signal West Pico Kit (Thermo Fisher Scientific Inc., Waltham, MA, USA). Protein levels were analyzed by Gel-Pro4.0 software (Media Cybernetics, Rockville, MD, USA).

### Immunofluorescence

In brief, cells were rinsed in PBS three times, fixed in 4% paraformaldehyde for 15 min and permeabilized in 0.1% Triton X-100 (Thermo Fischer Scientific, San Jose, CA) for 20 min. After three washes, cells were blocked in a 5% solution of bovine serum albumin (Sigma, San Antonio, USA) in PBS for 1 h at 37°C, followed by incubation with a 1:100 dilution of primary antibodies against vimentin, E-cadherin, N-cadherin or α-SMA at 4°C overnight. Cy3-labelled anti-rabbit IgG secondary antibody was used for visualization of specific signals under a fluorescent microscope. The nuclei of cells were counted after staining with DAPI-Fluoromount-G. All antisera were purchased from Bioworld Technology (Bioworld Technology, Inc, MN, USA).

### Enzyme-linked immunosorbent assay (ELISA)

ELISA was used to quantify concentrations of cytokines IL-10, TGF-β and IL-17 in each group. ELISA kits for the detection of each cytokine were obtained from R&D (R&D Systems, Minneapolis, MN). The assays were performed in duplicate with 50 µl of sample added to each well following the manufacturer's instructions. The readings were taken in an Epoch BioTek® ELX 800 plate reader (BioTek, Winooski, VT). The OD was read at 450 nm with reference to 630 nm. A standard curve was prepared for each cytokine, and the corresponding curve formulas were used to calculate the sample concentrations.

### Flow cytometry

Approximately 1×10^6^ lymphocytes in the culture group and the co-culture group were separately collected. CD4 and CD25 antibodies (Sigma, San Antonio, USA) were added to detect different subtypes of Treg cells. 4 °C in the dark for 30 min, wash with PBS twice; then add separately NRP1 or Foxp3 antibodies (Sigma), 4 °C in the dark for 30 min, wash with PBS twice; cells were centrifuged for 5 min at 1500 rpm, The solution was fixed with PBS. Then, the cells were analyzed by flow cytometry (FACScan, BD Biosciences). In addition, ICOS and CTLA-4 antibodies (Sigma) were directly added to the culture group and the co-culture group, and were incubated at 4 °C for 30 min, wash with PBS twice and analyzed using a flow cytometry.

### Cell migration assay

Cell migration assays were performed using 8 μm pore size 24-well transwell plate (Corning Inc). A549 cells were placed into the upper well of each chamber and the 3D cultured medium were added to the bottom chamber. After incubating for 48 h, the cells were fixed in 4% paraformaldehyde for 20 min, washed three times with PBS and stained with 1% Crystal violet (Solarbio, Beijing, China) for 20 min, cells located at upper chamber were cleaned out. Under the light microscopy, the number of migration cells was counted in five random regions (× 200) and averaged.

### Statistical analysis

The differences in expression of target proteins between groups were analyzed by Student's *t*-test. All statistical tests were performed with SPSS version 22.0 (SPSS, Inc., Chicago, USA).* p* < 0.05 was considered as statistically significant difference.

## Results

### Construction of the cell model

**Figure 1:**
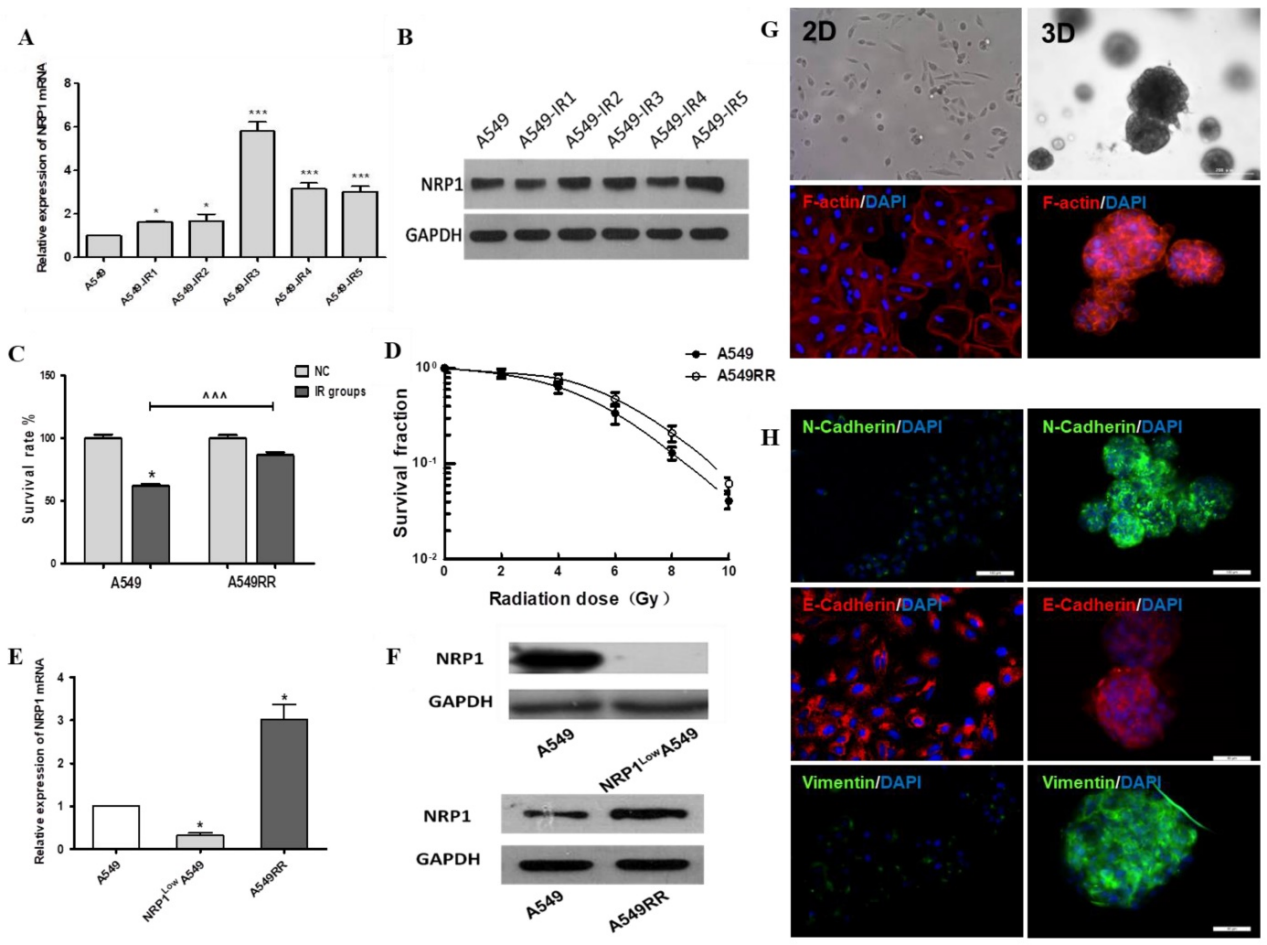
** Construction and analysis of the A549RR, NRP1^Low^A549 cell models and 3D-culture model.** Changes in NRP1 expression during construction of the A549RR model at the mRNA** (A)** and protein level **(B)**. Effect of 10 Gy X-ray irradiation on survival of A549RR cells **(C)** and colony forming ability **(D)**. NRP1 expression in the A549RR and NRP1^Low^A549 cell models at the mRNA **(E)** and protein level **(F)**. Changes in the morphological and cytoskeleton of A549 cells grown in Matrigel matrix were clearly distinguished by bright field conditions or phalloidin and DAPI staining: F-actin in red and DAPI-stained nuclei in blue **(G)**. Changes in the EMT-related marker proteins vimentin, N-cadherin and E-cadherin in 2D and 3D culture **(H)**. *p<0.05, ***p<0.001 *vs* control group; **^^^^^***p*<0.001 *vs* A549 IR group (n=3).

In the process of constructing the A549 radiation resistance (A549RR) cell model, we found that NRP1 was significantly elevated in protein and mRNA levels after multiple irradiations (Figure 1A-B). Thereafter, the detection of cell colony forming ability and cell viability showed that A549RR had stronger viability and colony forming ability after ionizing radiation than A549 cells (Figure 1C-D). These results provided preliminary confirmation that the A549RR model had been successfully created. Finally, we also verify the expression of NRP1 in three cell model. NRP1 mRNA in NRP1^Low^A549 cell was significantly lower than A549 cell (*p*<0.05), but A549RR cells increased significantly (*p*<0.05; Figure 1E), matched by corresponding changes in NRP1 protein levels (Figure 1F).

Under 2D conditions A549 cells formed monolayers and displayed spindle shapes with irregular growth. Compared with 2D culture, the cytoskeleton of A549 cells in 3D culture was more dense, the cells showed spherical growth with a wide range of connections and significant changes in cell morphology (Figure 1G). Using immunofluorescence detection of EMT-related protein markers, vimentin and N-cadherin expression were significantly increased in 3D culture compared to 2D-cultivated cells, and expression of E-cadherin was decreased (Fig. 1H); thus, the 3D-culture model may facilitate cell-matrix interactions in the tumor micro- environment and promote EMT transformation of tumor cells.

### Effect of NRP1 on microenvironment of tumor cells *in vivo*

**Figure 2:**
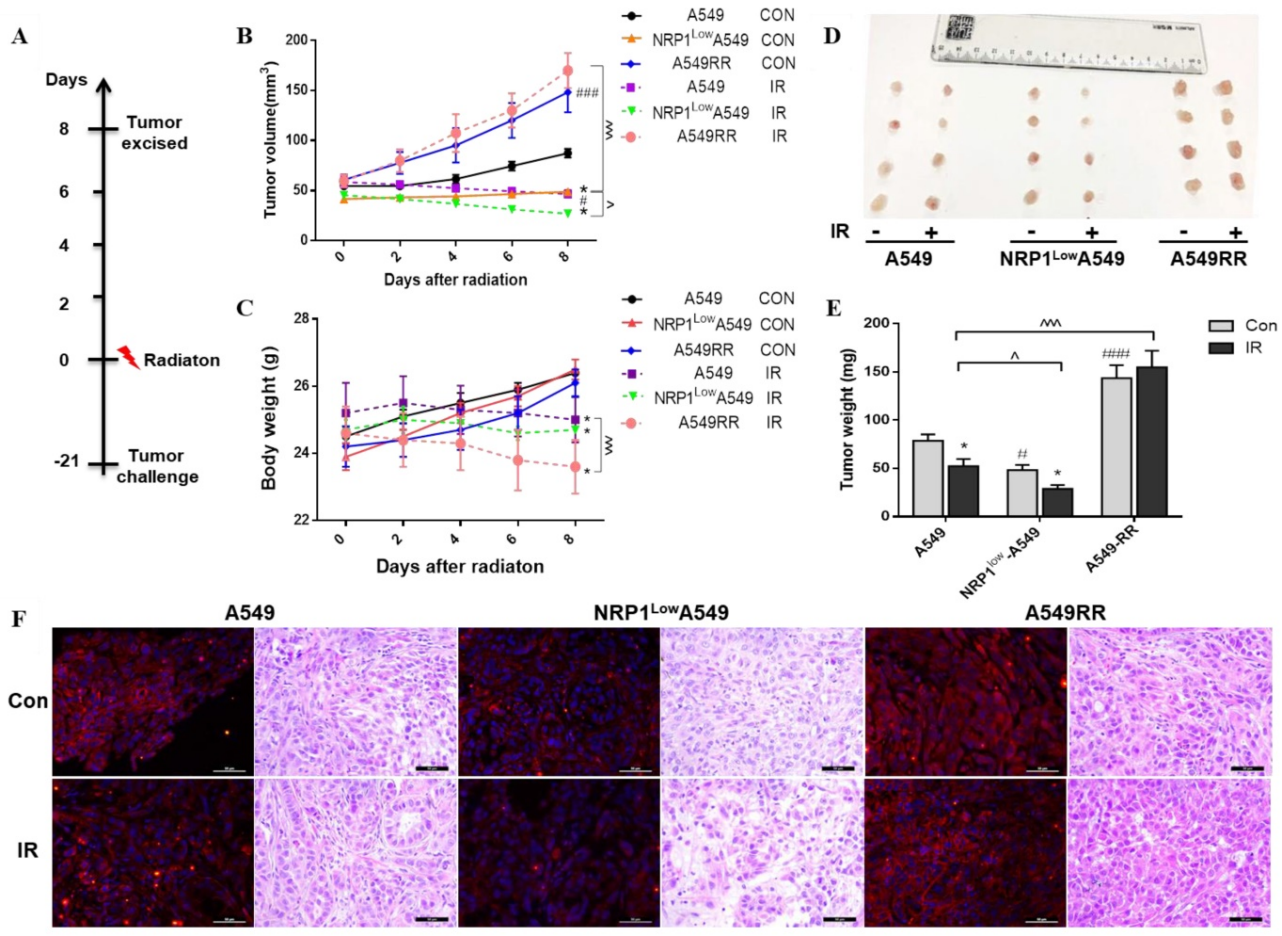
** Construction and analysis of the A549, NRP1^Low^A549 and A549RR mouse tumor model.** Establishment of the mouse tumor model and treatment regimen** (A).** Tumor volume over the course of treatment **(B)**. Body weights of mice in each group afther the radiaton treatment (n = 6/group). Data are expressed as the mean ± standard deviation. **(C).** Solid tumors excised from mice **(D)**. Tumor weight of each group **(E).** NRP1 expression and Histopathology photographs of the tumor tissues in the nude mice in the 6 groups **(F)**. **^#^***p*<0.05,**^###^***p*<0.001 *vs* A549 group; *p<0.05 *vs* control group; **^^^***p*<0.05,**^^^^^***p*<0.001 *vs* A549 IR group.

To examine the effecte of NRP1 in microenvironment of *in vivo* for tumor cell, we constructed the mouse tumor-bearing model and treatment of mice (Figure 2A). The tumor growth rate and tumor volume of each group were positively correlated with NRP1 expression. Notably, the tumor volume of A549 and NRP1^Low^A549 groups decreased after ionizing radiation (*p*<0.001), but there was no significant change in A549RR group (Figure 2B). The body weight of the mice without ionizing radiation in each experimental group was not significantly different. After irradiation, the body weight of each group decreased to different degrees, and the A549RR group was the most obvious (*p*<0.001; Figure 2C). It may be due to the effect of ionizing radiation and the size of the tumor. These observations were confirmed by an analysis of the tumor weight in each group (Figure 2D-E).

The expression of NRP1 in each group of tumor tissues was confirmed by immunofluorescence and found to be the same as *in vitro* (Figure 2F). Further by HE staining, the tumor cells of A549 group were arranged neatly and closely, and a large number of nuclei were observed. The number of nuclei in NRP1^Low^A549 group was significantly reduced, the arrangement was loose, and the mitotic phase was less. The tumor cells of A549RR group were clustered and clustered, the growth is strong, the shape and size are different. After irradiation, each group has different degrees of cancer cell damage, such as cytoplasmic condensation and nuclear pyknosis. Compared with the A549 group, the NRP1^Low^A549 group showed more extensive cell damage, a large number of apoptosis, and scattered isolated cells and large area necrotic areas. These results indicate that NRP1 potently enhances the proliferative capacity and radiation resistance of A549 cells *in vivo*.

### Effect of the tumor inflammatory microenvironment on NRP1-induced radiation resistance

**Figure 3:**
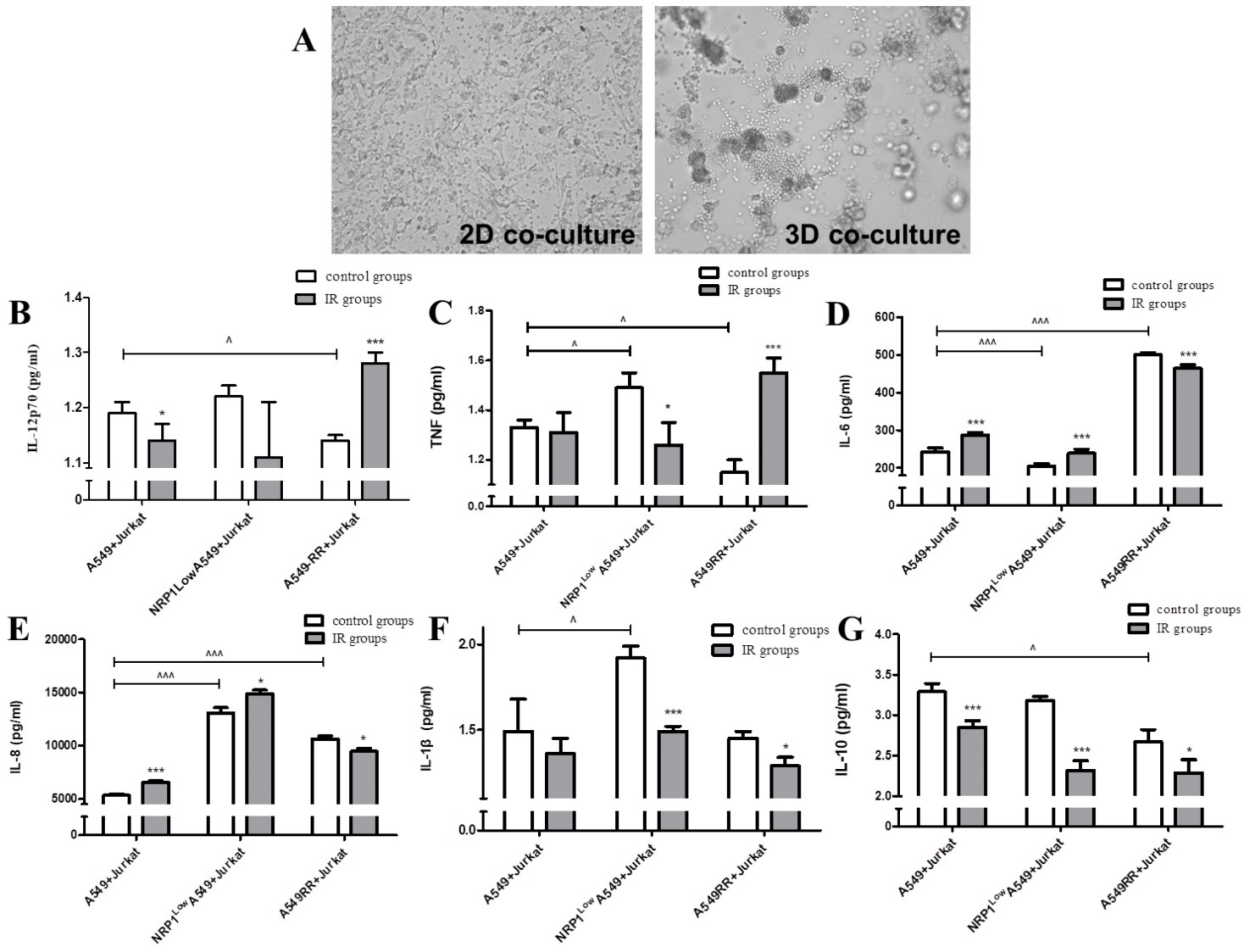
** Effect of the tumor inflammatory microenvironment on NRP1-induced lung cancer cell radiation resistance.** A549 and Jurkat cells were observed under bright field conditions in 2D and 3D co-culture for morphological changes **(A)**. The effect of NRP1 on the secretion of inflammatory factors in the inflammatory microenvironment of lung cancer: IL-12P70** (B)**; TNF **(C)**; IL-6** (D)**; IL-8** (E)**; IL-1 beta **(F)**; IL-10 **(G)**. *p<0.05, ***p<0.001 *vs* control group; ^*p*<0.05, ^^^*p*<0.001 *vs* A549 group (n=3).

Jurkat and A549 cells were co-cultured under 2D and 3D conditions, respectively, to reconstruct the tumor inflammatory microenvironment. In the 2D co-culture model, we observed that the two cell types were evenly distributed and the cytoskeleton did not change significantly. In the 3D co-culture model (Figure 3A), Jurkat cells were concentrated around the A549 cytoplasm and under Jurkat inflammatory action, the cytoplasm was somewhat broken and irregular. In the tumor inflammatory microenvironment mimicked by the 3D co-culture system, secretion of inflammatory cytokines IL-12p70 and TNF in the A549RR group was decreased compared with the A549 group (*p*<0.05; Fig. 3B-C). However, after irradiation with ionizing radiation, the opposite changes in expression were observed in these two groups; i.e., expression was increased in A549RR group (*p*<0.05) and decreased in the NRP1^Low^A549 group (*p*<0.05; Figure 3B-C). IL-6 and IL-8 expression in the A549RR group was significantly increased compared to the A549 group (*p*<0.001; Figure 3D-E), but differed in NRP1^Low^A549 group, where IL-6 expression was significantly reduced (*p*<0.001; Figure 3D), while IL-8 expression was significantly higher in both the NRP1^Low^A549 and A549RR groups (*p*<0.001; Figure 3E). After ionizing radiation, expression changes for both proteins in the two groups were similar to those observed before irradiation; i.e., expression was decreased in the A549RR group (*p*<0.05) but increased in the NRP1^Low^A549 group (*p*<0.05). The IL-1β and IL-10 expression levels in the two groups changed little, but their levels did decrease slightly after irradiation compared with non-irradiated cells (*p*<0.05; Figure 3F-G).

### The effect of ionizing radiation on the differentiation and function of Treg cells in the inflammatory microenvironment

To investigate the effects of ionizing radiation on immune tolerance in the tumor microenvironment, we constructed a 2D co-culture system of A549 cells and human peripheral blood lymphocytes, and detected the expression of costimulatory molecules and cytokines involved in Treg cell differentiation in the tumor inflammatory microenvironment. The results are shown in Table 1. In the co-culture group, the percentage of CD4^+^CD25^+^, CD4^+^CD25^+^FoxP3^+^ and CD4^+^CD25^+^NRP1^+^ Treg cells was significantly higher than in lymphocytes cultured alone (*p*<0.05), while after 10 Gy X-ray irradiation, with the exception of the CD4+CD25+ group, the percentage differences between the irradiated and control groups were not statistically significant. Inducible costimulator (ICOS) and cytotoxic T lymphocyte-associated antigen-4 (CTLA-4) expression in the co-culture groups was significantly higher than in single-culture cells (*p*<0.05). After 10 Gy X-ray irradiation, CTLA-4 expression was markedly increased compared with the control group (*p*<0.05), while ICOS did not show any significant change (Table 2). Compared to the single-culture groups, co-culture significantly increased IL-10 and TGF-β content in the supernatant (*p*< 0.05), but 6 Gy X-ray irradiation after co-culture failed to change the content of these proteins in either group. In contrast, IL-17 secretion was significantly higher in the co-culture group after irradiation compared to the non-irradiated co-culture control group and the irradiated single-culture group (*p*<0.05; Table 3).

### Effect of NRP1 on the expression of related cytokines in tumor inflammatory microenvironment *in vivo*

**Figure 4:**
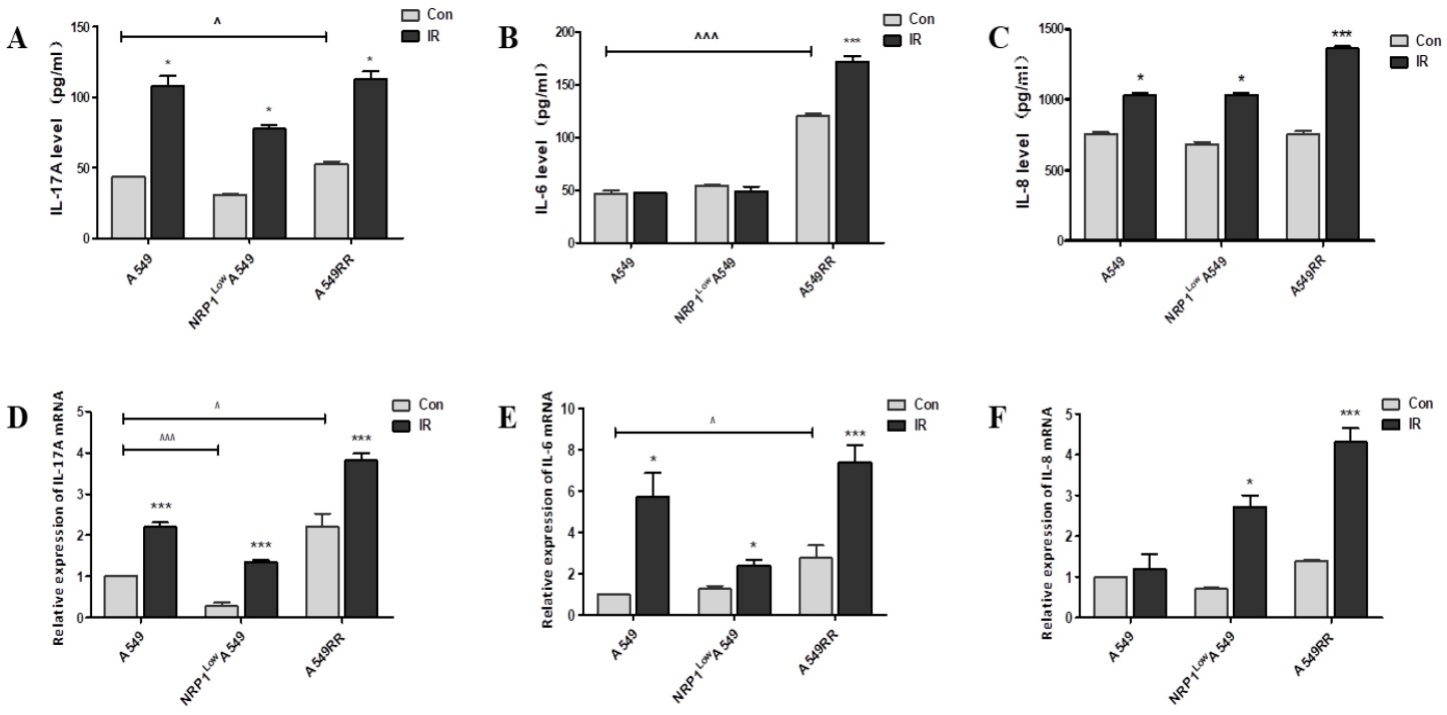
** Effect of NRP1 on IL-17A, IL-6, and IL-8 levels in the tumor tissue.** The tumor tissue of the mice in each group was taken and cytokine levels were analyzed by Elisa. IL-17A **(A)**, IL-6 **(B)**, IL-8 **(C)**; mRNA expression of inflammatory factors in tumor tissue were detected by qRT-PCR. IL-17A **(D)**, IL-6 **(E)**, IL-8 **(F)**; Data are presented as Mean ± SD (n=6). ^*^p<0.05, ^***^p<0.001 *vs* control group; ^^^*p*<0.05, ^^^^^*p*<0.001 *vs* A549 group (n=3).

In the above experiments, we examined the differentiation of Treg cells and the secretion of inflammatory cytokines in the tumor inflammatory microenvironment under 2D and 3D co-culture conditions, and found that NRP1-induced radiation resistance is associated with the expression of IL6, IL8 and IL-17A. Therefore, we further verified the effect of NRP1 on the secretion of these inflammatory factors at mRNA and protein levels in a tumor-bearing mouse model. Results are as follows, The high expression of NRP1 in the A549RR group boosted the mRNA and protein levels of IL-17A and IL-6 in tumor tissue, respectively. However, the low expression of NRP1 in the NRP1^Low^A549 group had little effect on the expression of the three factors, probably because their status was not activated. The expression of the three factors in each group increased with different degrees after ionizing radiation, and it was especially obvious in the A549RR group (Figure 4A-F). It can be seen that the radiation resistance induced by NRP1 is closely related to the expression of these three factors.

### Effect of the tumor migratory microenvironment on NRP1-induced lung cancer cell radiation resistance and migration

**Figure 5:**
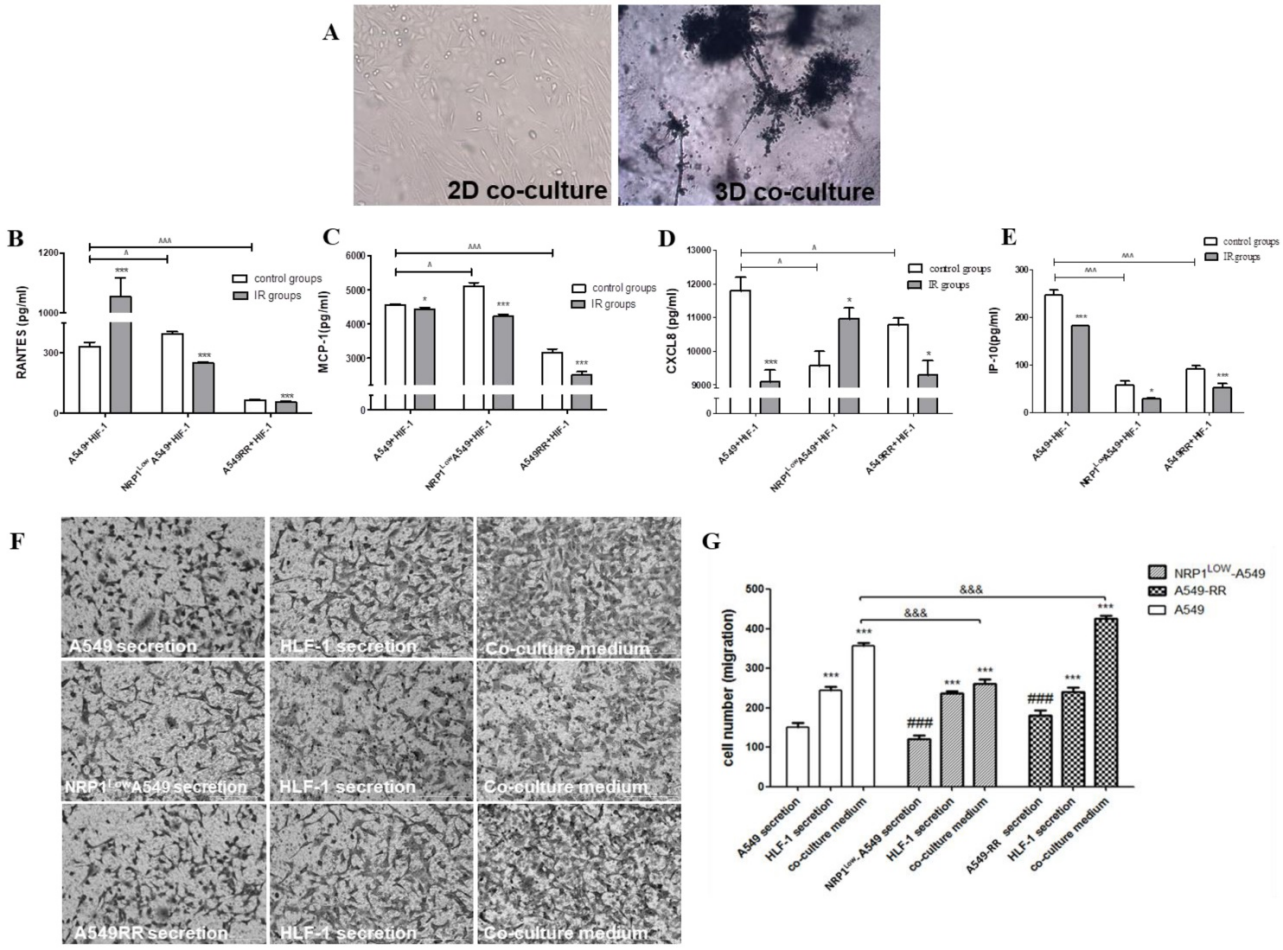
** Construction of the tumor migratory microenvironment and the effect of NRP1 on relevant chemokine secretion and migration of A549 cells.** A549 and HLF-1 cells were observed under bright field conditions for 2D and 3D co-culture morphological changes **(A)**. The effect of NRP1 on the secretion of chemokine factors in the migratory microenvironment of lung cancer cells: RANTES **(B)**; MCP-1 **(C)**; CXCL8 **(D)**; IR-10 **(E)**. *p<0.05, ***p<0.001 *vs* control group; ^^^*p*<0.05, ^^^^^*p*<0.001 *vs* A549 group (n=3). Effect of NRP1 on migration of A549 cells in the tumor migratory microenvironment **(F)**. ***p<0.01 vs control; ^###^*p*<0.01 vs A549 secretion; ^&&&^*p*<0.01 vs A549 co-culture (n=5).

In the 3D co-culture model formed by HLF-1 fibroblasts and A549 cells, HLF-1 cells obviously encapsulated A549 cells due to the chemotactic effect of fibroblasts which migrated to the distal end of larger cell spheres (Figure 5A). In the 3D co-culture system, changes in chemokine secretion in the tumor migratory microenvironment are shown in Figure 5B-F. Compared with the A549 group, RANTES and MCP-1 expression in the A549RR group decreased (*p*<0.05). RANTES and MCP-1 expression in the NRP1^Low^A549 group was significantly higher than expression in the control group (*p*<0.05). In contrast, IP-10 and CXCL8 expression were lower in all other groups except the A549 group and were significantly decreased in the NRP1^Low^A549 group (*p*<0.05). However, after ionizing radiation, CXCL8 expression in both groups displayed the opposite pattern of that seen before irradiation; i.e., expression was significantly decreased in the A549RR group (*p*<0.05) and was significantly increased in the NRP1^Low^A549 group (*p*<0.05). However, after exposure to ionizing radiation, IP-10 expression decreased in both groups compared with expression before irradiation (*p*<0.05).

The Transwell method was used to investigate the effect of tumor microenvironment on the migration of A549 cells in different states of NRP1 expression (Figure 5F-G). In A549RR cell supernatant, the number of migrating A549 cells significantly increased (*p*<0.01) compared with the other group. After co-culture, the tumor migratory microenvironment formed by inclusion of HLF-1 cells significantly promoted the migration of A549 cells when NRP1 was highly expressed, but when NRP1 was inhibited, the number of A549 cells that migrated was close to the number of migrating cells in the HLF-1 group cultured alone. Thus, the migration ability of tumor cells was closely related to NRP1 expression in the tumor microenvironment.

### Effect of NRP1 on the transformation of fibroblasts and EMT in tumor migration microenvironment

**Figure 6:**
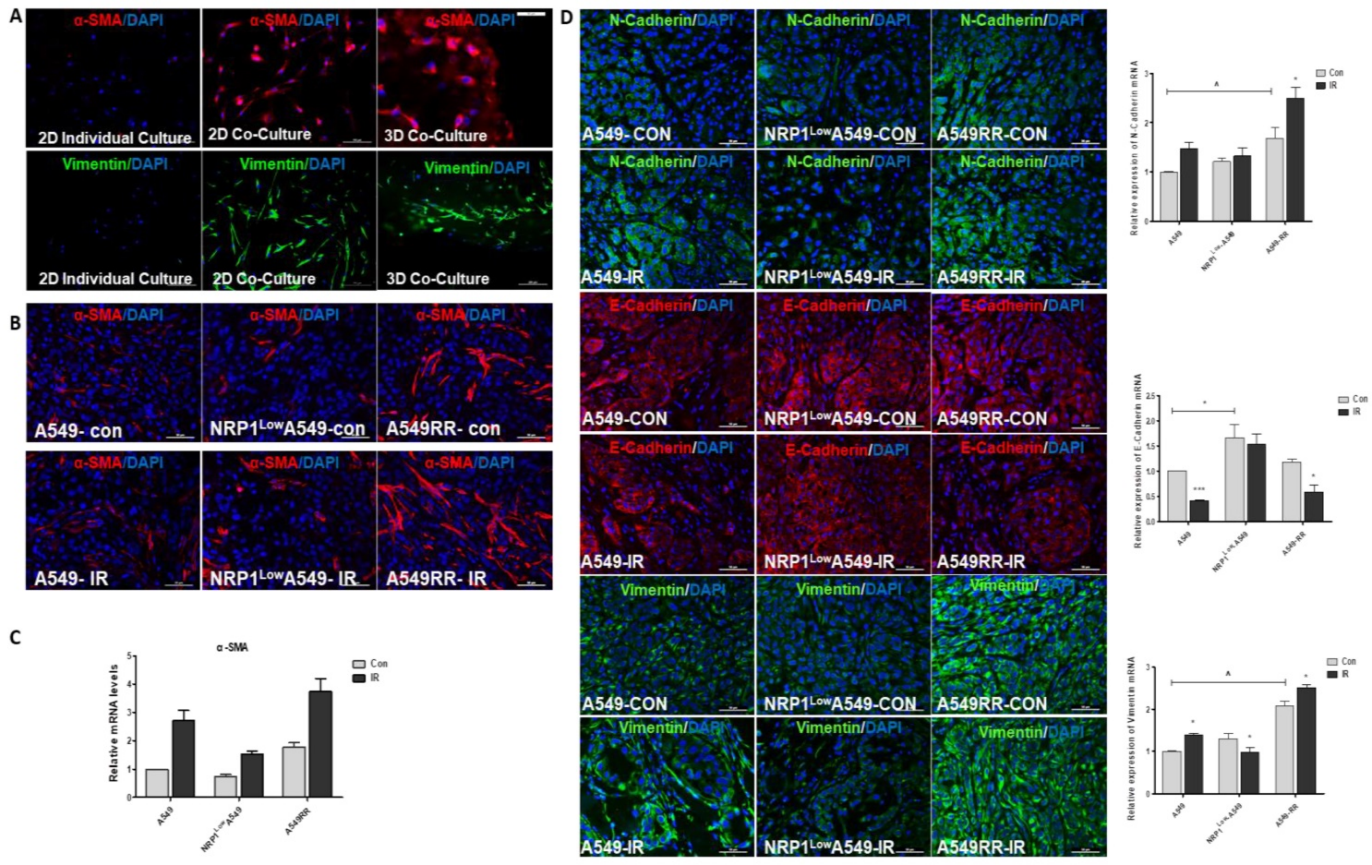
** Effect of NRP1 on the expression of tumor metastasis-related proteinsin in the tumor migratory microenvironment.** Changes in α-SMA and vimentin protein expression in HLF-1 cells in 2D and 3D co-culture systems **(A)**. The mRNAs levels of α-SMA in the HLF-1 cells **(B)**. Changes in α-SMA protein expression in tumor tissues **(C)** α-SMA mRNAs levels** (D)**. The protein expression of E-cadherin, N-cadherin and vimentin in the tumors of different groups. *p<0.05 *vs* control; & *p*<0.05 *vs* A549 group (n=3).

It has been reported in the literature that after co-culture of fibroblasts with tumor cells, fibroblasts are activated as CAFs, with increased expression of their marker proteins α-SMA and vimentin^18^. Therefore, we found that α-SMA and vimentin were increased in HLF-1 cells by 2D co-culture with A549 cells, and further enhanced under 3D co-culture conditions (Figure 6A). Since then we also verified this result in tumor-bearing tissues, and we found that after ionizing radiation, the number of fibroblasts transformed into CAF cells increased and was positively correlated with NRP1 expression (Figure 6B). And at the mRNA level, α-SMA was positively correlated with NRP1 expression (Figure 6C). We also verified whether EMT transformation of tumor cells itself in tumor tissues. From the results we found, the expression of EMT-related markers (E-cadherin, N-cadherin and vimentin) was positively correlated with NRP1, and under the action of ionizing radiation, NRP1 promoted EMT transformation of tumor cells, thereby enhancing the metastatic ability of tumor cells which in turn enhances radiation resistance (Figure 6D).

### Effect of NRP1 on TGF-β/Smad pathway in tumor migration microenvironment

**Figure 7:**
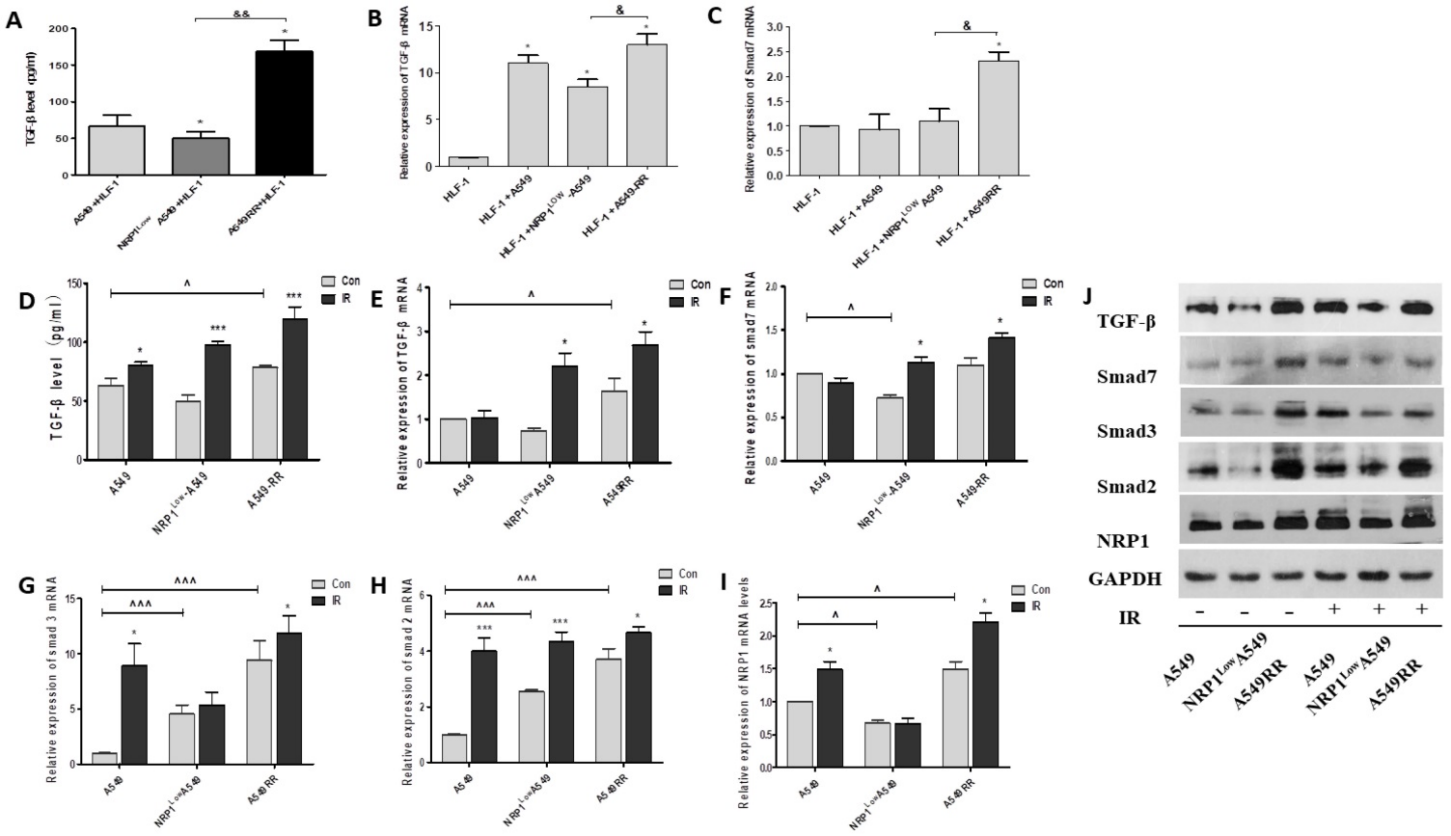
** Effect of NRP1 on TGF-β/Smad signaling pathway in the tumor migratory microenvironment.** The effect of NRP1 on the secretion of TGF-β in the migratory microenvironment. 2D co-culture systems **(A)**, In tumor tissue **(D)**; mRNA expression of TGF-β and smad7 in 2D co-culture systems **(B-C)**. qRT-PCR was performed to measure the regulatory effect of NRP1 on mRNA expression of TGF-β/Smad signaling pathway in tumor tissue. TGF-β **(E)**, Smad7 **(F)**, Smad3 **(G)**, Smad2 **(H)**, NRP1 **(I)**; Western blot was applied to investigate the regulation of NRP1 on TGF-β/Smad signaling pathway in tumor tissues **(J)**; *p<0.05 vs control; & p<0.05, && *p*<0.001 *vs* NRP1^Low^A549 co-culture group; **^** *p*<0.05, **^^^** *p*<0.001 *vs* A549 con group (n = 3).

As is known, TGF-β plays key regulatory role on Smads signaling pathway, and it is necessary to investigate whether NRP1 could promote the EMT through TGF-β mediated Smads signaling pathway. We examined the effect of NRP1 in the 2D co-culture system and found that TGF-β secretion and NRP1 expression were positively correlated (*p*<0.05). That is, the expression of TGF-β and Smad7 was significantly increased in the A549RR co-culture group, but significantly lower in the NRP1^Low^A549 co-culture group than in the A549RR co-culture group (Figure 7A-C). Afterwards, we further verified this in tumor-bearing tissues. In the A549RR group, the protein and mRNA levels of TGF-β and Smas2/3 were significantly higher than those of the other two groups, and further increased after ionizing radiation (Figure 7D-J). These data further implied that NRP1 might promoted EMT through regulating the TGF-β/Smads signaling pathway in the tumor migratory microenvironment.

## Discussion

The tumor microenvironment is a special and complex system, a 3D structure composed of a variety of cells and matrix which is regulated through complex signaling pathways inducing a variety of proinflammatory cytokines, chemokines and angiogenesis factors to promote tumor development 19. Multicellular 3D culture and interaction with stromal components are considered essential elements in establishing a 'more clinically relevant' tumor model. Recently some report conducted to reconstruct the tumor inflammatory or migratory microenvironment by co-culture of tumor cells with immune cells or fibroblasts, and to further study the impact of the tumor microenvironment on tumor progression 19. So in this study, we established a 2D or 3D co-culture system to mimic the *in vivo* microenvironment, and then further validated the results by constructing a mouse tumor-bearing model *in vivo*. To elucidate the immune tolerance of lung cancer cells in the tumor microenvironment and tumor migration mechanisms in the migratory microenvironment after the action of ionizing radiation, and further explored the mechanisms mediating the effect of the tumor microenvironment on NRP1-induced radiation resistance.

In our experiments, an experimental method developed in our laboratory was used to build an A549RR cell model by multiple exposures to high doses of X-ray irradiation. The results suggested that radiation resistance was related to the increase in NRP1. It was thus confirmed again that NRP1 could induce radiation resistance in lung cancer cells. For the 3D culture environment, the pressure state and contact mode between cells is changed 22. Our results showed that after 3D culture, the morphology of A549 cells changed significantly, the cytoskeleton contracted, and the cells became closely linked, forming a 3D gradient of multicellular groups, which is more similar to growth *in vivo*. And the EMT process in A549 cells and the expression of related proteins changed significantly after 3D culture. This confirmed that the 3D culture system can mimic characteristics of the tumor *in vivo* and replicate the clinical features of tumor-triggering EMT to promote tumor cell invasion and migration. Therefore, we were able to use the 3D co-culture system to simulate the tumor microenvironment and further study the mechanisms of tumor cell proliferation, invasion and metastasis.

In our study, we used tumor-bearing model *in vivo* for confirming the role of NRP1 in tumor microenvironment around the cancer cells. We found that NRP1 decreased lung cancer cell apoptosis and death in mouse tumor-bearing model after the action of ionizing radiation and that the effect was similar when compared to *in vitro*. As we hypothesized, NRP1 played critical roles in tumor growth and cell radiation resistance, respectively. Therefore, we further explored the role of NRP1 in the tumor inflammatory microenvironment produced under 3D co-culture conditions. The results found that, in our experiments the high TNF secretion level was inhibited in the A549RR group. Comparing the effect of ionizing radiation on the A549RR and A549 groups, TNF secretion increased significantly in A549RR group. In the inflammatory microenvironment of the tumor, TNF may exert an anti-tumor effect by influencing the expression of p53 protein through binding of surface-specific receptors on tumor cells 23. And the TNF secretion was shown to increase significantly after ionizing radiation in a dose-dependent manner 24. Therefore, NRP1 induced resistance to high radiation via a synergistic effect on TNF. It is also known that IL-6 can activate the STAT3 pathway in the tumor microenvironment, induce a large number of inflammatory genes and further promote angiogenesis to alter the proliferation of tumor cells 25. Tamatani et al. 26 found that ionizing radiation can increase the expression of IL-8 and inhibit the activity of NF-κB, which can reduce the expression of some inflammatory factors including IL-8 and enhance the radiosensitivity of tumors. Our results showed that under 3D co-culture conditions and tumor-bearing tissues, IL6 expression in A549RR group was higher than that of the other two groups before and after irradiation. Compared with the control group, IL-6 and IL-8 expression increased in the NRP1^Low^A549 group after irradiation. This may be due to the stimulation of IL-6 secretion in the tumor microenvironment, which in turn affects immune tolerance. This indicates that in the process of radiation-induced lung cancer resistance induced by NRP1, both inflammatory reactions and the radiosensitivity of lung cancer cells may be reduced by regulating IL-6 and IL-8 in the tumor inflammatory microenvironment.

We also employed 2D co-culture conditions to analyze the effects of ionizing radiation on the differentiation of Treg cells and the expression of related factors in the tumor microenvironment. Known for T cell proliferation and killing ability, induced Treg cells are involved in tumor escape, tolerance and other processes 27. We showed that the proportion of CD4^+^CD25^+^ Tregs in lymphocytes increased significantly after co-culture, and Foxp3, NRP1 and CTLA-4 expression on the surface of the cells were upregulated, indicating that the tumor cells stimulated T cell immune responses. The stimulation of CD4^+^CD25^+^ Tregs and the expression of CTLA-4 after 10 Gy ionizing radiation can further enhance immunosuppression and promote the occurrence and development of tumors. The experimental results also showed that IL-17 secretion did not change significantly in the 2D co-culture system and each group of unirradiated tumor-bearing tissues, but after exposure to irradiation, IL-17 was significantly increased, suggesting that the effect of ionizing radiation can promote immune tolerance in the tumor microenvironment and increase radiation resistance in tumor cells. Therefore, in the tumor inflammatory microenvironment, ionizing radiation effects on NRP1 may regulate IL-17, TNF, IL-6, IL-8 and other inflammatory factors to enhance the radiation resistance of A549 cells. Differentiation and expression of related cytokines induced by Treg cells may thus promote immune tolerance in the tumor inflammatory microenvironment.

In terms of the tumor migratory microenvironment, MCP-1 as chemokine has an important influence on angiogenesis, migration and the invasion ability of tumor cells^28^. Research has found that the high RANTES expression activates the signaling pathways of phosphatidylinositol 3-kinase, Akt, IKKα/β and NF-κB, which in turn enhances the migration ability of lung cancer cells 29. In our experiments, we found that NRP1 could effectively inhibit the secretion of MCP1 and RANTES in the tumor migratory microenvironment, after irradiation, the secretion of MCP1 and RANTES showed a further decrease. Studies have also shown an effect of IP-10 on tumor microenvironment (TME) in natural killer (NK) cell migration and adhesion function 30. The CXCL8 and IP-10 exert anti-tumor effects in the tumor migratory microenvironment 31. Our results also confirmed this effect and showed that the expression in the A549RR group was significantly lower than the A549 group, and was reduced to varying degrees after irradiation. Additional studies on changes in migration ability of lung cancer cells under different migration microenvironments have found that the migration ability of cells is closely related to NRP1 expression in the migration microenvironment. These results suggest that NRP1 induces radiation resistance and regulates the secretion of related chemokines such as IP-10 and CXCL8 in the microenvironment surrounding tumor metastases, which in turn influences the activation status of relevant signaling pathways and enhances the migration ability of tumor cells and it may enhance tumor cell radiation resistance.

According to the results of cell migration in the tumor migration microenvironment, it is necessary to further explore whether the changes in tumor cell metastasis function are related to the transformation of major cells in the microenvironment. It is known that fibroblasts differentiate into CAFs under the action of TGF-β are key fibrogenic factors and play an important role in the regulation of α-SMA protein and mRNA expression in CAFs. And the Smad protein signaling pathway is involved in TGF-β-induced differentiation of lung fibroblasts into myofibroblasts 32. TGF-β could bind and phosphorylate cell-surface receptors (TGF-βRI/ TGF-βRII), and the activated TGF-βRI phosphorylates Smad2 or Smad3 will subsequently bind to Smad4. The Smad complex moves into the nucleus and interacts with various transcription factors to regulate the transcription of downstream genes 33. Our results showed that TGF-β secretion was positively correlated with NRP1 expression and related factors (α-SMA, Smad7) in fibroblasts. In addition, we detected fibroblasts in 2D or 3D co-culture models and tumor tissues, found that α-SMA and vimentin protein expression was significantly increased, indicating that some cells transformed into CAFs. Tirino et al. found that, after TGF-β stimulation, the A549 cells showed the EMT performance, and the cells invasion and metastasis signifcantly enhanced 34. The occurrence of EMT involved the regulation of many genes, and several studies have implied the importance of E-cadherin and vimentin in regulating EMT 35, 36. *In vivo*, we further demonstrated that NRP1 can enhanced EMT through regulating the TGF-β/Smads signaling pathway, which can regulating the expression of E-cadherin, N-cadherin and vimentin, promote the EMT process, and finally elevate the ability of A549 cells metastasis.

In summary, 2D, 3D co-culture methods and mouse tumor-bearing model was established to mimic the inflammatory and migratory microenvironments of lung cancer, and simulated the growth state of lung cancer cells in a manner more similar to the actual *in vivo* situation. The effects of intercellular interactions, and interactions of cells and extracellular matrix were explored to determine the impact of NRP1 on the inflammatory and migratory microenvironment of lung cancer cells and to elucidate its mechanism of action in radiation resistance. Our hope is to provide a new theoretical and experimental basis for radiation therapy in clinical lung cancer.

## Supplementary Material

Supplementary table S1.

## Figures and Tables

**Table 1:** The effect of ionizing radiation on the differentiation of Treg cells in the microenvironment of lung cancer cells ‾(x±s, %).

Group	CD4^+^ CD25^+^			CD4^+^ CD25^+^ FoxP3^+^			CD4^+^ CD25^+^ NRP1^+^	
	Control	IR		Control	IR		Control	IR
**Individual culture group**	16.15 ± 1.02	20.05 ± 2.78^		3.71 ± 0.53	3.74 ± 0.68		3.36 ± 0.40	4.65 ± 2.22
**Co-culture group**	28.60 ± 3.75*	33.75 ±1.13*^		17.56 ± 0.55*	16.37±1.44*		13.27 ± 3.05*	13.89 ± 1.13*

*p<0.05 *vs* individual culture group; ^*p*<0.05 *vs* control group; n=3

**Table 2:** Effect of ionizing radiation on ICOS and CTLA-4 expression in the microenvironment of lung cancer cells (x±s, %).

Group	ICOS			CTLA-4	
	Control	IR		Control	IR
**Individual culture group**	1.79 ± 1.26	1.72 ± 1.41		2.29 ± 0.95	4.63 ± 0.69^
**Co-culture group**	7.62 ± 1.23*	9.14 ± 1.87*		10.05±1.26*	13.21 ± 2.21*^

*p<0.05 *vs* individual culture group; ^*p*<0.05 *vs* control group; n=3

**Table 3:** Effect of ionizing radiation on IL-10, TGF-β and IL-17 secretion in the microenvironment of lung cancer cells (x±s, ng/ml).

Group	IL-10		TGF-β		IL-17	
	Control	IR	Control	IR	Control	IR
**Individual culture group**	15.9±2.5	12.3±3.3	6579.4±470.2	6116.9±580.8	22.0±10.9	24.3±11.1
**Co-culture group**	207.7±27.1*	202.0±41.7*	9276.9±590.4*	9382.2±764. *	24.5±6.4	54.1±12.7*^

*p<0.05 *vs* individual culture group; ^*p*<0.05 *vs* control group; n=3

## References

[1] Siegel RL, Miller KD, Jemal A (2017). Cancer Statistics, 2017. CA Cancer J Clin.

[2] Ferlay J, Soerjomataram I, Dikshit R (2015). Cancer incidence and mortality worldwide: sources, methods and major patterns in GLOBOCAN 2012. Int J Cancer.

[3] Uzel EK, Abacioglu U (2015). Treatment of early stage non-small cell lung cancer: surgery or stereotactic ablative radiotherapy?. Balkan Med J.

[4] Nagaraja SS, Krishnamoorthy V, Raviraj R (2017). Effect of Trichostatin A on radiation induced epithelial-mesenchymal transition in A549 cells. Biochem Biophys Res Commun.

[5] Chang L, Graham PH, Hao J (2014). Emerging roles of radioresistance in prostate cancer metastasis and radiation therapy. Cancer Metastasis Rev.

[6] Zachary I (2014). Neuropilins: role in signalling, angiogenesis and disease. Chem Immunol Allergy.

[7] Dong JC, Gao H, Zuo SY (2015). Neuropilin 1 expression correlates with the Radio-resistance of human non-small-cell lung cancer cells. J Cell Mol Med.

[8] Palodetto B, Da SSDA, Rodrigues LM (2017). SEMA3A partially reverses VEGF effects through binding to neuropilin-1. Stem Cell Res.

[9] Zahn LM (2017). Effects of the tumor microenvironment. Science.

[10] Wang M, Zhao J, Zhang L (2017). Role of tumor microenvironment in tumorigenesis. J Cancer.

[11] Li Y, Wan YY, Zhu B (2017). Immune Cell Metabolism in Tumor Microenvironment. Adv Exp Med Biol.

[12] Lyssiotis CA, Kimmelman AC (2017). Metabolic Interactions in the Tumor Microenvironment. Trends Cell Biol.

[13] Schiavoni G, Gabriele L, Mattei F (2013). The tumor microenvironment: a pitch for multiple players. Front Oncol.

[14] Thoma CR, Zimmermann M, Agarkova I (2014). 3D cell culture systems modeling tumor growth determinants in cancer target discovery. Adv Drug Deliv Rev.

[15] Alemany-Ribes M, Semino CE (2014). Bioengineering 3D environments for cancer models. Adv Drug Deliv Rev.

[16] Regier MC, Montanez-Sauri SI, Schwartz MP (2017). The Influence of Biomaterials on Cytokine Production in 3D Cultures. Biomacromolecules.

[17] Livak KJ, Schmittgen TD (2001). Analysis of relative gene expression data using real-time quantitative PCR and the 2(-Delta Delta C(T)) Method. Methods.

[18] Shan T, Chen S, Chen X (2017). Prometastatic mechanisms of CAF-mediated EMT regulation in pancreatic cancer cells. Int J Oncol.

[19] Salvatore V, Teti G, Focaroli S (2017). The tumor microenvironment promotes cancer progression and cell migration. Oncotarget.

[20] Salvatore V, Teti G, Bolzani S (2014). Simulating tumor microenvironment: changes in protein expression in an *in vitro* co-culture system. Cancer Cell Int.

[21] Jeong SY, Lee JH, Shin Y (2016). Co-Culture of Tumor Spheroids and Fibroblasts in a Collagen Matrix-Incorporated Microfluidic Chip Mimics Reciprocal Activation in Solid Tumor Microenvironment. Plos One.

[22] Swayne TC, Lipkin TG, Pon LA (2009). Live-cell imaging of the cytoskeleton and mitochondrial-cytoskeletal interactions in budding yeast. Methods Mol Biol.

[23] Cooks T, Harris CC (2014). p53 mutations and inflammation-associated cancer are linked through TNF signaling. Mol Cell.

[24] Pal S, Yadav P, Sainis KB (2016). TNF-alpha and IGF-1 differentially modulate ionizing radiation responses of lung cancer cell lines.

[25] Hamada S, Masamune A, Yoshida N (2016). IL-6/STAT3 Plays a Regulatory Role in the Interaction Between Pancreatic Stellate Cells and Cancer Cells. Dig Dis Sci.

[26] Tamatani T, Azuma M, Motegi K (2007). Cepharanthin-enhanced radiosensitivity through the inhibition of radiation-induced nuclear factor-kappaB activity in human oral squamous cell carcinoma cells. Int J Oncol.

[27] He D, Li H, Yusuf N (2010). IL-17 promotes tumor development through the induction of tumor promoting microenvironments at tumor sites and myeloid-derived suppressor cells. J Immunol.

[28] Dong JC, Gao H, Zuo SY (2015). Neuropilin 1 expression correlates with the Radio-resistance of human non-small-cell lung cancer cells. J Cell Mol Med.

[29] Swamydas M, Ricci K, Rego SL (2013). Mesenchymal stem cell-derived CCL-9 and CCL-5 promote mammary tumor cell invasion and the activation of matrix metalloproteinases. Cell Adh Migr.

[30] Fu X, Yang B, Lao S (2013). Human memory-like NK cells migrating to tuberculous pleural fluid via IP-10/CXCR3 and SDF-1/CXCR4 axis produce IFN-gamma in response to Bacille Calmette Guerin. Clin Immunol.

[31] Yoshio T, Okamoto H, Kurasawa K (2016). IL-6, IL-8, IP-10, MCP-1 and G-CSF are significantly increased in cerebrospinal fluid but not in sera of patients with central neuropsychiatric lupus erythematosus. Lupus.

[32] Ji Y, Dou YN, Zhao QW (2016). Paeoniflorin suppresses TGF-beta mediated epithelial-mesenchymal transition in pulmonary fibrosis through a Smad-dependent pathway. Acta Pharmacol Sin.

[33] Geng J, Fan J, Ouyang Q (2014). Loss of PPM1A expression enhances invasion and the epithelial-to-mesenchymal transition in bladder cancer by activating the TGF-beta/Smad signaling pathway. Oncotarget.

[34] Tirino V, Camerlingo R, Bifulco K (2013). TGF-beta1 exposure induces epithelial to mesenchymal transition both in CSCs and non-CSCs of the A549 cell line, leading to an increase of migration ability in the CD133+ A549 cell fraction. Cell Death Dis.

[35] Chen PC, Lee WY, Ling HH (2018). Activation of fibroblasts by nicotine promotes the epithelial-mesenchymal transition and motility of breast cancer cells. J Cell Physiol.

[36] Yu H, Shen Y, Hong J (2015). The contribution of TGF-beta in Epithelial-Mesenchymal Transition (EMT): Down-regulation of E-cadherin via snail. Neoplasma.

