# Supplementary material for: The Role of the Tumor Microenvironment in Neuropilin 1-Induced Radiation Resistance in Lung Cancer Cells
Source: J Cancer. 2019 Jul 8;10(17):4017–30. doi: 10.7150/jca.28163 (PMC6692609; doi:10.7150/jca.28163)
Supplement: Supplementary file 1 — Supplementary table S1. [file jcav10p4017s1.pdf]

Supplementary Table 1. Primer for quantitive Real-time PCR

Primer name	Sequence(5'-3')	Sequence(3'-5')
**GAPDH**	ACATCGCTCAGACACCATG	TGTAGTTGAGGTCAATGAAGGG
**NRP1**	CCCCAAACCACTGATAACTCG	AGACACCATACCCAACATTCC
**α-SMA**	TGGTGGGAATGGGACAAAA	CGTGAGCAGGGTGGGATG
**TGF-β**	TGGTGGAAACCCACAACGAA	AGAAGTTGGCATGGTAGCCC
**Smad2**	ATGTCGTCCATCTTGCCATTC	AACCGTCCTGTTTTCTTTAGCTT
**Smad3**	CACGCAGAACGTGAACACC	GGCAGTAGATAACGTGAGGGA
**Smad7**	TCCTGCTGTGCAAAGTGTTC	TTGTTGTCCGAATTGAGCTG
**IL-17A**	AACCGATCCACCTCACCTTG	TCTCTTGCTGGATGGGGACA
**IL-8**	ACTCCAAACCTTTCCACCCC	ATGAATTCTCAGCCCTCTTCAAA
**IL-6**	ACTCACCTCTTCAGAACGAATTG	CCATCTTTGGAAGGTTCAGGTTG
**E-cadherin**	CAGGTCTCCTCATGGCTTTGC	CTTCCGAAAAGAAGGCTGTCC
**N-cadherin**	AGCGCAGTCTTACCGAAGG	TCGCTGCTTTCATACTGAACTTT
**vimentin**	CGTCCACACGCACCTACAG	GGGGGATGAGGAATAGAGGCT
